# Training zones through muscle oxygen saturation during a graded exercise test in cyclists and triathletes

**DOI:** 10.5114/biolsport.2023.114288

**Published:** 2022-06-01

**Authors:** Aldo A. Vasquez Bonilla, Adrián González-Custodio, Rafael Timón, Alba Camacho-Cardenosa, Marta Camacho-Cardenosa, Guillermo Olcina

**Affiliations:** 1 Faculty of Sport Sciences. University of Extremadura, Cáceres, Spain

**Keywords:** Near-infrared spectroscopy, Muscle oxygenation, Fatmax, Oxygen consumption, Performance, Cycling

## Abstract

Use of muscle oxygen saturation (SmO_2_) has been validated as a performance factor during incremental exercise with portable near-infrared stereoscopy (NIRS) technology. However, there is little knowledge about the use of SmO_2_ to identify training zones. The objective of this study was to evaluate the metabolic zones by SmO_2_: maximum lipid oxidation zone (Fatmax), ventilatory thresholds (VT1 and VT2) and maximum aerobic power (MAP) during a graded exercise test (GXT). Forty trained cyclists and triathletes performed a GXT. Output power (W), heart rate (HR), oxygen consumption (VO_2_), energy expenditure (kcal/min) and SmO_2_ were measured. Data were analysed using the ANOVA test, ROC curves and multiple linear regressions. Significance was established at p ≤ 0.05. SmO_2_ decreases were observed from baseline (LB) to Fatmax (*Δ* = -16% p < 0.05), Fatmax to VT1 (*Δ* = -16% p < 0.05) and VT1 to VT2 (*Δ* = -45% p < 0.01). Furthermore, SmO_2_ together with weight, HR and output power have the ability to predict VO_2_ and energy expenditure by 89% and 90%, respectively. We conclude that VO_2_ and energy expenditure values can be approximated using SmO_2_ together with other physiological parameters and SmO_2_ measurements can be a complementary parameter to discriminate aerobic workload and anaerobic workload in athletes.

## INTRODUCTION

Graded exercise tests (GXT) to exhaustion are traditionally used to evaluate the performance in cyclists through the evaluation of physiological parameters such as heart rate (HR), central venous oxygen saturation (ScvO_2_), oxygen consumption (VO_2_), ventilatory equivalent (VEQ) and power (W) [1, 2]. Generally, these parameters provide performance information from the athlete so as intensity increases, different training zones can be determined by the participation of metabolic pathways or energy [3]. Knowing this process is necessary to schedule individual workouts and can help optimize performance in endurance athletes. However, there are limitations to the detection of subtle changes in other physiological factors that affect performance and changes in metabolic pathways – for example, oxygenation changes in skeletal muscle, which cannot be determined with a cardiopulmonary gas analyser during GXT in endurance athletes [4, 5].

In studies of skeletal muscle physiology, near infrared stereoscopy (NIRS) technology is commonly used to detect changes in muscle oxygenation during exercise [6] and provides new knowledge about the muscle oxidative capacity [7]. Likewise, in the latest reviews on the use of NIRS as a research tool in sports settings, it has been shown that portable NIRS has good sensitivity to detect skeletal muscle oxygen supply and use during static and dynamic muscle work in response to interventions in exercise and training [8].

The Monitor portable NIRS device can evaluate the muscle oxygen saturation (SmO_2_). For example, Moxy, which is affordable in price in the consumer market, it has been validated in comparison with other NIRS brands [9]. But, according to Crum [10], Moxy seems to have proven reliable to measure SmO_2_ at low and moderate intensities during a GXT, but not at high intensities, where greater variation has been observed. However, Crum’s study did not examine whether Moxy was able to discriminate between different training zones through the metabolic pathways used by skeletal muscle [11]. This would be interesting for future studies using other brands of portable NIRS.

By evaluating the training zones based on changes in muscle metabolism we can identify: the maximum lipid oxidation zone (Fatmax), ventilatory thresholds VT1 and VT2 and maximum aerobic power (MAP) [12, 13, 14]. With the information from these four zones, we can program workouts with different objectives. Currently heart rate (%) and VO_2_ (l/m) are the most used internal load parameters in cycling [15] (Swain & Leutholtz, 1997) but including the measurement of SmO_2_ is interesting because it allows the evaluation of muscle oxidative metabolisms and the relationship with factors that limit exercise tolerance in cyclists and triathletes [16]. As far as we know, no study has yet evaluated SmO_2_ by training zones based on muscle metabolism in highly trained athletes, so the objectives of this study are, first, to evaluate SmO_2_ during a GXT and establish cut-off points for each training zone based on the scale (0%–100%), and second, to study the variables associated with ${\dot{\text{V}}\text{O}}_{2\max}$ using an explanatory model that incorporates SmO_2_ with other physiological parameters to identify the training zone.

## MATERIALS AND METHODS

### Subjects

The sample was composed of 40 trained cyclists and triathletes (age: 32 ± 8 years; height: 180 ± 005 cm; body mass: 74.6 ± 7.6 kg; ${\dot{\text{V}}\text{O}}_{2\max}$: 64.6 ± 7.3 ml/kg/min; HR rest: 45.3 ± 6.3; % body fat: 9.6 ± 2.4; skinfold of the vastus lateralis: 13.4 ± 7.9 mm and experience in endurance training: 10.9 ± 4.9 years) who volunteered to participate in this study. The subjects were healthy individuals without any physical limitations or muscular-skeletal injuries that might affect the outcome of the exercise test. The study was carried out in accordance with the Helsinki Declaration and approved by the Bio-Ethical Committee of the University of Extremadura with the registration code: XXXX. Signed consent was obtained from each subject prior to their participation.

### Trial design

The trial design was cross-sectional. Subjects carried out the test under similar environmental conditions (21–24ºC and 45–55% relative humidity) and were asked to abstain from doing intense exercise 48 hours prior to the test. Before the test, the anthropometric variables were determined: weight, percentage body fat [17] and vastus lateralis fold. The GXT was then carried out to obtain SmO_2_.

### Maximal graded exercise test (GXT)

First, a standardized warm-up of 10 min at 100 W was performed; the ramp protocol consisted of increments of 30 W · min^-3^ until volitional exhaustion [13]. The end of the test was considered when the participant was unable to maintain the power output of each final completed stage. During GXT participants were monitored through a gas exchange measurement system/device with breath-by-breath technology and calibrated before each test (Metalyzer 3b, CORTEX Biophysik GmbH, Leipzig, Germany). Each participant used their own bike mounted on a smart training device (Bkool, model Bkool one; Madrid, Spain). The protocol was completed with a PowerTap P1 (PP1), which produced reliable output power readings of 100–500 W, in a seated position (rho ≥ 0.987), and an absolute reliability index (150–500 W; COV = 2.3%; SEM < 1.0 W) [18]. The PowerTap during cardiopulmonary tests are more ecologically valid, allowing cyclists to use their own bicycles [18]. HR was collected via a HR monitor (HRM-Tri; Garmin Ltd., Olathe, KS, USA). The smart trainer assessed power with internal sensors that were paired to a smartwatch for future analysis (Forerunner 735xt, Garmin, Olathe, KS, USA).

### Muscle oxygen saturation assessment

For the evaluation of local muscle oxygen saturation (SmO_2_) a near-infrared spectroscopy device with a sampling frequency of 1 Hz (MOXY, Fortiori Design LLC, Minneapolis, Minnesota, USA), which is valid for measuring SmO_2_ (ICC: r = 0.773–0.992), was used [10]. It was attached firmly to the belly of the right vastus lateralis (VL) muscle (12 cm above the patella border muscle) using a dark elastic strap to avoid light contamination and movement artefacts. The vastus lateralis was selected based on previous evidence and considering the role of this muscle in cycling [19]. The skinfold thickness at the NIRS measurement site VL was measured using a skinfold caliper (Harpenden Ltd.) to ensure that the skinfold thickness was < 1/2 of the distance between the emitter and the detector (25 mm). Furthermore, no participant presented values > 15 mm from the skinfold, as a reference point where SmO_2_ measurements are usually excluded [20]. This technology allows the evaluation of saturation taking into consideration the relative change of total haemoglobin (tHb) and the interaction between O_2_Hb and HHb with the following equation:

$${\text{SmO}}_{2}=\left(\left({\text{O}}_{2}\text{Hb})/({\text{O}}_{2}\text{Hb}+\text{HHb}\right)\right)\times 100.$$

This is calculated by the quantification of variation in optical transmission by sequentially emitting light waves (630–850 nm) from light diodes into the tissue and recording the amount of light received. Using an algorithm, the system determines the amount of light absorbed at wavelengths relative to the oxygenated and deoxygenated Hb using the Beer-Lambert Law and tissue light propagation model processes. The raw muscle O_2_ saturation (SmO_2_) signal was treated with a soft spline filter to reduce noise created by movement [21] using Minitab 19 (Minitab, Inc, State College, PA; www.minitab.com, USA) (see Supplementary figure).

For the data analysis the following guidelines were followed: 1) the mean values of the last minute of each phase were used 2) exclude data of SmO_2_ > 10% after the last recorded value and 3) data that gave a 0% reading were excluded due to the apparent lost signal. The data were viewable in real time to the NIRS technology expert researchers and muscle oxygenation measurement, using ANT + technology software (Golden Cheetah version 3.4, USA) and joint data processing software (Excel 2016, Microsoft Office 365, USA).

### Gas exchange analysis of VT1 and VT2 by V-slope method and independent experts

**FIG. 1:**
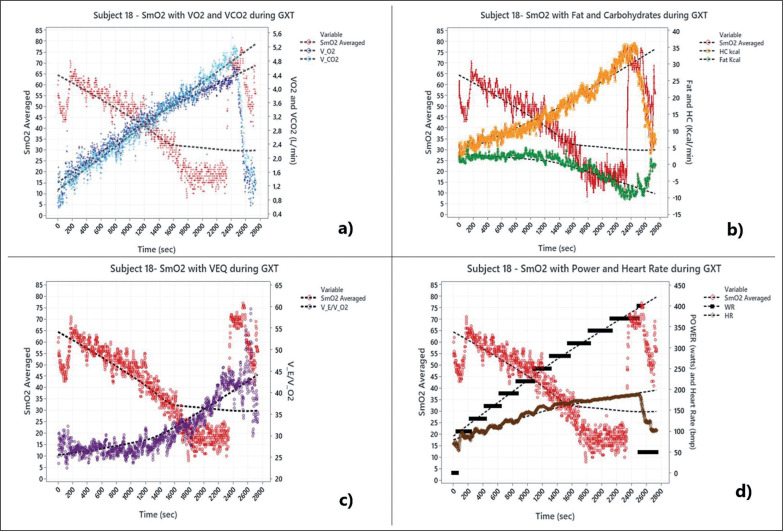
Measurement of muscular oxygen saturation with other physiological parameters during maximal graded exercise test (example of a participant in this study).

The thresholds provided by MetaSoft Studio were adopted and confirmed according to the criteria of the expert researchers. VT1 and VT2 were determined with the equivalent ventilatory method (VEQ). VT1 was determined using the criteria of an increase in ventilatory equivalent of oxygen (VE/VO_2_) with no concomitant increase in ventilatory equivalent of carbon dioxide (VE/VCO_2_) [22]. VT2 was determined using the criteria of an increase in both the VE/VO_2_ and VE/VCO_2_ (Figure 1d) and analysing the behaviour of VCO_2_ as a function of VO_2_, during progressive exercise tests when exceeding the lactate thresholds is accompanied by the buffering of lactic acid by [HCO_3_] with a consequent VCO_2_ increase. This results in a transition in the relationship between VCO_2_ and VO_2_, which is the underlying element in all anaerobic threshold methods of detection by gas exchange [23, 24]. V-Slope load was identified in that intensity of exercise which, in a plot of the minute production of CO_2_ over the minute utilization of oxygen (VO_2_), shows an increase in the slope above 1.0 [23, 25] (Figure 1a). The ${\dot{\text{V}}\text{O}}_{2\max}$ was defined as the highest plateau (two successive maxima within 150 mL · min^-1^, averaging the data every 5 s) reached [26].

### Fatmax zone

The fat oxidation (FO) and carbohydrate oxidation (CHO) were calculated using appropriate stoichiometric equations [27] and energy equivalence, based on the

$$\begin{array}{l} \text{FAT}(\text{g}/\text{min})=\left[\left(1.67\times {\text{VO}}_{2})-(1.67\times {\text{VCO}}_{2}\right)\right]\\ \text{CHO}(\text{g}/\text{min})=\left[\left(4.55\times {\text{VCO}}_{2})-(3.21\times {\text{VO}}_{2}\right)\right]\\ \text{FAT}(\text{kcal}/\text{min})=\left[\left(1.67\times {\text{VO}}_{2})-(1.67\times {\text{VCO}}_{2}\right)\right]\times 9\\ \text{CHO}(\text{kcal}/\text{min})=\left[\left(4.55\times {\text{VCO}}_{2})-(3.21\times {\text{VO}}_{2}\right)\right]\times 4 \end{array}$$

Data analysis to determine Fatmax involved the measured values approach in the stage with the highest recorded fat oxidation value and the corresponding ${\dot{\text{V}}\text{O}}_{2}$ [13] (Figure 1 b).

### Statistical analysis

A descriptive analysis of the data extracted from the incremental test was applied and the Shapiro-Wilk normality test was applied for each variable. When normality was reached, the one-way repeated ANOVA test was performed for comparison of each step and training zone and then a Tukey’s b post hoc test was applied to identify the internal differences between the steps. In addition, we observed the percentage change between each stage. Statistics based on the magnitude of the differences were applied to determine their clinical significance (standard deviation for individual response; SD_IR_). SD_IR_ values were appraised against a minimum clinically important difference (MCID = SEM × 1.96 × √2) of each stage. Meaningful interindividual differences were observed in the ability to decrease SmO_2_ considered present for a given variable when the SDIR exceeded the MCID (Yes) [28]. For results where the MCID exceeded the SD of the next stage (w), we reverse the SD_IR_ formula and report these instances as negative SD_IR_ values (No). Using such an approach allows us to make comparisons between the different stages, where the data shifted from classifying individuals based on their measured change scores to classifying the change scores themselves in order to identify the interindividual response [29]. Receiver operating characteristic (ROC) curves were then used to establish a cut-off for SmO_2_ in each training zone, and the area under the curve (AUC) was used to evaluate the performance of the test, where the threshold cut-off values were defined by the points representing the highest concomitant sensitivity and specificity. The AUC was interpreted according to the following guidelines: not informative (AUC = 0.5), less accurate (0.5–0.7), moderately accurate (0.7–0.9), high precision (0.9–1) and perfect discriminatory test (AUC = 1.0). Finally, the Pearson correlation test was used to study the relationship of physiological parameters and a multiple linear regression analysis was performed among the variables associated with generating prediction equations of VO_2_ and energy expenditure through SmO_2_. The level of significance was established at p < 0.05 with 95% confidence intervals. The results were expressed as mean ± standard deviation. All analyses were performed with SPSS software (version 22).

## RESULTS

Table 1 gives a description of the physiological parameters obtained from the GXT for each step. A progressive decrease in SmO_2_ was observed, as expected, but without significant changes. The %HR reserve, VO_2_ (ml/kg/min for l/min), carbohydrate oxidation/carbohydrate energy and the total energy expenditure (kcal/min) increased during the test. No inter-individual response was identified as positive.

**TABLE 1:** Description of physiological parameters by step during the graded exercise test in cyclists and triathletes.

Steps / Variables	3′	6′	9′	12′	15′	18′	21′	24′	27′	30′	33′
Power (watts)	100 ± 4	130 ± 6	160 ± 7	190 ± 9	220 ± 13	250 ± 8	280 ± 9	310 ± 8	340 ± 7	370 ± 6	400 ± 34

Heart Rate (ppm)	110 ± 14	118 ± 15	127 ± 15	136 ± 16	148 ± 19	152 ± 13	161 ± 12	168 ± 12	174 ± 13	180 ± 15	181 ± 13

Heart Rate Reserve (%)	47 ± 7	53 ± 8	60 ± 8*	66 ± 9*	74 ± 10*	78 ± 7	85 ± 6	89 ± 5	94 ± 4	96 ± 2	96 ± 3

Oxygen Consumption (ml/kg/min)	23.4 ± 3.3	26.9 ± 3.5	31. 3 ± 4.2*	35.8 ± 4.5*	40.7 ± 5.5*	43.7 ± 4.1	48.0 ± 4.2	52.1 ± 4.7	56.1 ± 5.5	59.4 ± 6.7	63.4 ± 7.3

Oxygen Consumption (L/min)	1.73 ± 0.25	1.99 ± 0.22	2.31 ± 0.23*	2.64 ± 0.25*	3.00 ± 0.32*	3.26 ± 0.32	3.58 ± 0.37*	3.88 ± 0.36	4.17 ± 0.35	4.39 ± 0.43	4.70 ± 0.46

Muscle Oxygen Saturation (%)	60 ± 8	56 ± 10	51 ± 11	44 ± 13	35 ± 15	32 ± 13	26 ± 11	21 ± 10	20 ± 8	17 ± 6	16 ± 4

Fat Oxidation Rate (g/min)	0.33 ± 0.19	0.35 ± 0.19	0.41 ± 0.21	0.42 ± 0.24	0.38 ± 0.26	0.36 ± 0.28	0.34 ± 0.29	0.25 ± 0.20	0.20 ± 0.19	-	-

Carbohydrate Oxidation Rate (g/min)	1.4 ± 0.5	1.7 ± 0.6	2.0 ± 0.6*	2.4 ± 0.7	2.9 ± 0.8	3.4 ± 1.0*	3.8 ± 0.8	3.9 ± 0.5	5.1 ± 0.7*	-	-

Fat energy (Kcal/min)	3.0 ± 1.7	3.2 ± 1.7	3.7 ± 1.9	3.8 ± 2.1	3.4 ± 2.4	3.2 ± 2.5	3.0 ± 2.6	2.3 ± 1.8	1.9 ± 1.7	-	-

Carbohydrate energy (Kcal/min)	5.6 ± 2	6.8 ± 2.2	7.8 ± 2.2*	9.5 ± 2.7	11.7 ± 3.2	13.7 ± 3.9*	15.1 ± 3.3	15.4 ± 1.9	20.4 ± 2.8*	-	-

Total Energy (Kcal/min)	8.6 ± 1.3	10 ± 1.2*	11.5 ± 1.2*	13.3 ± 1.3*	15.1 ± 1.6*	16.9 ± 2.0	18.1 ± 1.9	19.7 ± 1.7	22.7 ± 2.1*	-	-

**Inter-individual response (SmO_2_%)** **SD_IR_ > (MCID)**		**14% (7%)** **NO**	**16% (8)** **NO**	**16 (9)** **NO**	**17 (11)** **NO**	**19 (11)** **NO**	**17 (9)** **NO**	**15 (8)** **NO**	**12 (7)** **NO**	**9 (5)** **NO**	**6 (4)** **NO**

N° subjects	N = 40	N = 40	N = 40	N = 40	N = 40	N = 40	N = 40	N = 33	N = 27	N = 14	N = 6

Note:

* p ≤ .0.05 compared to the previous stage. SD_IR_ = standard deviation of individual responses, MCID = minimum clinically important difference. Qualitative interpretations of SD_IR_ > SWC are provided for results with positive SD_IR_ values.

Table 2 shows the differences by training zone during the exercise from baseline to the end of the test. Changes in SmO_2_ were identified from BL to Fatmax (Δ = 9.0 p = 0.017 MCID = 8%), Fatmax to VT1 (Δ = 8.6, p = 0.024 MCID = 10%) and VT1 to VT2 (Δ = 19.5 p = 0.000 MCID = 9%). However, there is no significant decrease after VT2, so SmO_2_ did not detect changes in MAP (Δ = 5.6 p = 0.404). Similarly, there was no positive interindividual response from VT2 to PAM. Likewise, changes were identified in power (%), HR (ppm), heart rate reserve (%), VO_2_ (ml/kg/min) and VO_2_ (l/min) in all training zones.

**TABLE 2:** Differences of physiological parameters by training zones during the graded exercise test in cyclists and triathletes.

Training Zones / Variables	Baseline	∆ (%)	Fatmax	∆ (%)	VT1	∆ (%)	VT2	∆ (%)	MAP (${\dot{\text{V}}\text{O}}_{2\max}$)
Power (W)	100 ± 0	62%*	162 ± 43	23%*	200 ± 41	45%**	290 ± 41	21%*	352 ± 49
Power (%)	27 ± 0	62%*	44 ± 10	23%*	55 ± 10	45%**	81 ± 8	21%*	98 ± 7
Heart Rate (ppm)	110 ± 14	14%*	126 ± 16	10%*	139 ± 13	20%*	167 ± 12	7%*	180 ± 12
Heart Rate Reserve (%)	47 ± 7	23%*	58 ± 8	15%*	67 ± 7	31%*	88 ± 3	10%*	97 ± 2
Oxygen Consumption (ml/kg/min)	23.4 ± 3.3	34%*	31 ± 6.2	19%*	37 ± 6.2	35%**	50 ± 7	16%*	58.4 ± 7.4
Oxygen Consumption (L/min)	37 ± 5	29%	48 ± 7	18%*	57 ± 6	38%**	79.0 ± 6	13%*	90 ± 5
Muscle Oxygen Saturation (%)	60 ± 9	-16%*	50 ± 12*	-16%*	42 ± 13	-45%**	23 ± 10	-26%	17 ± 7

**Inter-individual response (SmO_2_%)** **SD_IR_ > (MCID)**			**12% (8)** **Yes**		**17% (10)** **Yes**		**18% (9)** **Yes**		**12% (7)** **NO**

Note:

* p < 0.05 and

** p < 0.01 compared to the previous stage. Subject numbers (n = 40). SD_IR_ = standard deviation of individual responses. MCID = minimum clinically important difference. Qualitative interpretations of SD_IR_ > SWC are provided for results with positive SD_IR_ values. Training Zones = Maximum lipid oxidation (Fatmax), Ventilatory thresholds (VT1 and VT2) and Maximal Aerobic Power (MAP).

**FIG. 2:**
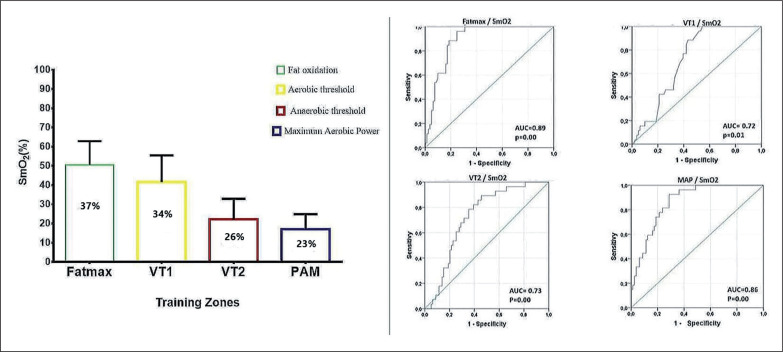
Analysis of the receiver operating characteristics (ROC) curve to evaluate the cut-off for muscle oxygen saturation for each training zone.

Figure 2 represents the ROC curve for SmO_2_-based training zones. The areas under the curve (AUC) were found in SmO_2_: Fatmax: ≤ 37% (AUC); sensitivity = 0.891 and specificity = 0.247; SE = 0.30; IC = 0.831 to 0.950 (equivalent: 42%–36%); p = 0.000, VT1: ≤ 34% (AUC); sensitivity = 0.718 and specificity = 0.370; SE = 0.48; IC = 0.824 to 0.813 (equivalent: 35%–30%); p = 0.01, VT2: ≤ 26% (AUC); sensitivity = 0.786 and specificity = 0.354; SE = 0.50; IC = 0.175 to 0.371 (equivalent: 32%–23%); p = 0.000 and the MAP zone: ≤ 23% (AUC); sensitivity = 0.852 and specificity = 0.288; SE = 0.35; IC = 0.070 to 0.218 (equivalent: 28%–21%); p = 0.000.

Table 3 shows the most suitable variable equations for the multiple linear regression model that can be predicted by SmO_2_ as an alternative to a VO_2_ analyser. Our results indicated a high prediction in percentage 88% VO_2_ (l/min) 89% VO_2_ (ml/kg/min) and 90% total energy expenditure (kcal/min). The relevant equations are shown below.


$${\text{VO}}_{2}(\text{l}/\text{min})=-0.58-(0.007*{\text{SmO}}_{2})+(0.008+\text{W})+(0.003*\text{HR})+(0.022*\text{weight})$$ (1)

$${\text{VO}}_{2}(\text{ml/kg/min})=31.42-(0.09*{\text{SmO}}_{2})+(0.10*\text{W})+(0.05*\text{HR})-(0.25*\text{weight})$$ (2)

$$\text{Energy total}(\text{kcal}/\text{min})=-7.3-(0.015*{\text{SmO}}_{2})+(0.05*\text{W})+(0.02*\text{HR})+(0.13*\text{weight})$$ (3)

**TABLE 3:** Prediction Equations between muscle oxygen saturation with oxygen consumption and energy expenditure.

First order equation: Multiple linear regression								

Independent variable	Dependent variable	Non-standardized coefficient B	Standardized coefficient B	Correlation (r)	Ajusted R-Square	SEE	F Value	Sig.
**VO**_2_ **(L/min)**	(k)	-0.579 ± 0.226						
	SmO_2_ (%)	-0.007 ± 0.002	-0.146					
	Power (W)	0.008 ± 0.000	0.734	0.943	0.888	0.366	517.7	0.000
	Heart Rate (ppm)	0.003 ± 0.001	0.091					
	Weight (Kg)	0.022 ± 0.003	0.189					

**VO**_2_ **(ml/kg/min)**	(k)	31.417 ± 3.766						
	SmO_2_ (%)	-0.091 ± 0.025	-0.135					
	Power (W)	0.104 ± 0.007	0.716	0.939	0.880	5.087	479.2	0.000
	Heart Rate (ppm)	0.053 ± 0.018	0.114					
	Weight (Kg)	-0.247 ± 0.037	-0.153					

**Energy Total (kcal/min)**	(k)	-7.3 ± 1.392						
	SmO_2_ (%)	-0.015 ± 0.010	-0.060						
	Power (W)	0.048 ± 0.003	0.772	0.949	0.900	1.341	383.8	0.000
	Heart Rate (ppm)	0.022 ± 0.007	0.122					
	Weight (Kg)	0.126 ± 0.014	0.226					

## DISCUSSION

To the best of our knowledge, this is the first study to evaluate SmO_2_ based on training zones established by cut-off points and also to present equations that can be used by trained cyclists. During GXT, SmO_2_ progressively decreased as exercise intensity increased. However, no differences were observed in the change for each step (Table 1), which means that using a Fatmax protocol [13] where the steps go from 3 min to changes of 30 W no differences can be identified. This is similar to Crum’s study [10], where SmO_2_ changes were observed in each step increase (5 and 50 W), until the final stages of the test. Therefore, changes in SmO_2_ are only observable with large step differences in watts and by training zone (Table 2), except after passing VT2, where oxygen becomes independent for power production, and there was a change of metabolic pathway due to high intensity work. Furthermore, the Crum study indicates that at high intensities, SmO_2_ measured with a portable Moxy is not sensitive to changes. This can be observed in many studies that use Moxy [10, 30], but adequate treatment of the data is needed, such as the application of the soft spline filter (Figure 1 supplementary) [21], the Moxy calibration scale (0%–100%) [20] and the measurement standards as they were used in this study. Through such treatment, we were able to narrow down the problem. Similarly, studies have not reported measurement problems using this methodology [31, 32]. The contribution of our study is that it presents the difference of work in VT1 and VT2 with SmO_2_.

Another explanation for the lack of changes of SmO_2_ passing the VT2 is the data interpretation, because the behaviour of SmO_2_ at high intensity is non-linear, contrary to the moderate intensity zones, where it does retain linear behaviour [33]. This occurs due to changes in the metabolic pathway in the muscle where the regeneration of PCr in the last stages is limited by time and maximum power. There are also increases in blood flow due to the accumulation of vasoactive metabolites, such as H +, K + and lactate. That interrupts the use of energy to withstand fatigue in the high intensity zone [34, 35] Likewise, in our study, muscle oxygenation in cyclists and triathletes seemed to be affected at high intensities (VO_2_ = > 50 ml/kg/min). According to Oueslati et al. [36], when ${\dot{\text{V}}\text{O}}_{2\max}$ values are higher than this, anaerobic metabolism and lactate accumulation acquire greater relevance, causing an imbalance between oxygen supply and demand. Along the same lines, another factor to highlight in this study is that the greatest observable changes in SmO_2_ occur in the transition from VT1 to VT2, in line with two previous studies [37, 38] showing that the greatest differences in muscle oxygenation could be found when the VO_2_ values are between VT1 and VT2 (VO_2_ values > 40 ml/min/kg) [39]. This suggests the potential of SmO_2_ to observe an aerobic workload vs an anaerobic workload.

Likewise, although it is commonly believed that it is difficult to identify training zones with SmO_2_, in our study it was possible to establish cut-off points for each training zone. It was observed that in the VT2 and MAP zones the sensitivity and specificity were higher (Figure 2). This means that for values below < 26% it is more likely that the anaerobic energy system is being used. Compared with zones such as Fatmax and VT1, the specificity is lower; however, the change from higher to lower sensitivity and specificity is due to the physiological behaviour of oxygen-dependent and oxygen-independent exercise [40], that is, the change in energy pathway from VT1 to VT2. So far, this study proposes that new ways of assessing performance in high-intensity zones should be explored through non-linear changes, for example analysis of results with exponential and quadratic regressions; this is the next challenge for researchers in this line of work. Likewise, it is well known that Fatmax zone and muscle deoxygenation kinetics are associated [41], SmO_2_ remains stable in the presence of fat oxidation and at the SmO_2_ breakpoint where VT2 is entered one no longer consumes fat and is totally dependent on carbohydrate energy (Figure 1b). This is also a result of the subjects with better performance in endurance tests maintaining higher SmO_2_ values during exercise than sprinting athletes [42].

Regarding the interpretation of the predictive capacity in studies that use muscle oxygen, There is still none with the SmO_2_ variable, but we found the study by Montero et al. [43] which related the Hb mass in the muscle as an independent predictor of the VO_2_ peak when factoring in the regression analysis model (adjusted r^2^ = 0.73, P < 0.0001). Also, to predict VO_2_ and energy expenditure in our study, we used an equation and percentage prediction (r^2^ = 0.89 p < 0.000) using the SmO_2_ for HR, power and weight. Contrary to the ACSM predictive metabolic equations that only use the individual’s power and weight [44], however, our equations require SmO_2_ as a predictive metabolic marker of fats and carbohydrates (Figure 1b) and HR as a necessary cardiovascular parameter in the explanation of muscle oxygen extraction [45]. This makes it more specific to find the VO_2_ and energy expenditure. Additionally, with the cut-off points we can approximate the SmO_2_ values to VT1; this is observable when the VEQ has an increase in the VE/VO_2_ ratio without an increase in the VE/VCO_2_ ratio and an abrupt decrease in SmO_2_ occurs. VT2 corresponds to an increment of the VE/VCO_2_ ratio and a fractional decrease in the concentration of CO_2_ (PetCO_2_), and VT2 can be identified as a plateau of SmO_2_ (Figure 1 and 2). However, care must be taken, because this is the first time that SmO_2_ has been used.

Likewise, Paquette et al. [46] highlighted the importance of using peripheral adaptations during short and long events, since they were able to predict performance through muscle oxygenation by multiple regression analysis. That is, muscle oxygen extraction with NIRS was a predictor of metabolic performance that approximated VO_2_ and energy expenditure [46, 47]. Furthermore, using the set of SmO_2_ with HR provides information on the internal load of each athlete, because although there is an inverse correlation between HR and SmO_2_ (-0.71) [10], at high intensities, HR (> 90%) stabilizes and shows small changes. SmO_2_ continues to decrease slowly and then increases again at this precise moment due to the increase in the oxidative phosphorylation and the increase in blood flow caused by the hyperaemia response [48, 49]. This phenomenon is also known as critical oxygenation and was recently disregarded by Feldmann [50]. Also, power decreases, and then performance does as well. Finally, our equation is justified by the SmO_2_ values as an indicator of the internal load of metabolic demands [10], HR as an indicator of cardiovascular stress of the autonomic nervous system [51], as well as power as an indicator of the external load and weight as an indicator of the health factor of each athlete. All of these data are more accessible for athletes to obtain in each training session, if they wish to know the changes in the metabolic pathways at work without fatigue as VT1 vs working with more fatigue at VT2, using a portable NIRS (SmO_2_). We thus demonstrate the dependence of a better VO_2_ and a higher energy expenditure with these variables. These equations can be entered into software or a smartphone application, thus providing an alternative for coaches and athletes who do not have access to a cardiopulmonary test.

## LIMITATIONS

One of the limitations of this study is that the zones and performance points such as critical power, respiratory compensation point, and functional thresholds power were not defined, as these depend on muscle bioenergetics, although they approximate to VT2 by 21% [52]. Likewise, it would be interesting to observe the verification of VT1 and VT2 in submaximal efforts with intensity according to the determined threshold value of SmO_2_ (34 and 26%) with simultaneous measurements of blood lactate concentration. Furthermore, we have shown scientific advances for SmO_2_ measured with Moxy that were not studied in the Crum validation study. Moxy is an instrument that is not the most suitable for measuring SmO_2_ but is accessible to the athletic population.

## CONCLUSIONS

In conclusion, SmO_2_ measured with a portable NIRS can be a complementary physiological parameter to identify the transition from a more aerobic workload to a more anaerobic workload, because it is similar to indicating threshold changes from VT1 to VT2 during exercise. In addition, SmO_2_ at moderate intensity before entering VT1 can be interpreted as work of maximal fat oxidation (Fatmax). Finally, SmO_2_ together with power, heart rate and weight can predict performance by approximating VO_2_ and energy expenditure.
